# Epidemiological factors associated with HBV infection and uptake of testing in south west region of Cameroon: What can be done to scale up HBV testing in our setting?

**DOI:** 10.1371/journal.pgph.0000321

**Published:** 2022-05-11

**Authors:** Henry Dilonga Meriki, Kukwah Anthony Tufon, Teuwafeu Denis Georges, Ngomba Divine Martin Mokake, Ronald Mbua Gobina, Nyeke James Tony, Tebit Emmanuel Kwenti, Ayah Flora Bolimo, Malika Esembeson, Nkouonlack Cyrille, Fokam Djike Puepi Yolande, Iyah Rebecca Itoe, Tsamul Beltine, Theresa Nkuo-Akenji, Damian Nota Anong

**Affiliations:** 1 Department of Microbiology and Parasitology, University of Buea, Buea, South West Region, Cameroon; 2 Buea Regional Hospital, Buea, Southwest Region, Cameroon; 3 Department of Allied Health, Biaka University Institute, Buea, Southwest Region, Cameroon; 4 Department of Public Health and Hygiene, Faculty of Health Science, University of Buea, Buea, South West Region, Cameroon; 5 Department of Internal Medicine and Paediatrics, Faculty of Health science, University of Buea, Buea, South West Region, Cameroon; 6 Department of Surgery, Faculty of Health Sciences, University of Buea, Buea, Cameroon; 7 Department of Medical Laboratory Science, Faculty of Health Science, University of Buea, Buea, South West Region, Cameroon; 8 Department of Biological Science, Faculty of Science, University of Bamenda, Bamenda, North West Region, Cameroon; Universidad de Las Americas, ECUADOR

## Abstract

Hepatitis B infection affects millions of people globally, partly due to its high degree of transmissibility and asymptomatic nature. This study was aimed at identifying prevailing epidemiological factors associated with HBV infection and testing uptake in the South West region of Cameroon. This hospital-based case-control study enrolled HBV infected participants and “healthy” controls ≥18 years old. Venous blood collected from participants was used to conduct HBV panel test (HBsAg, anti-HBs, HBeAg, anti-HBe, anti-HBc). Data on demographic and behavioral risk factors as well as reasons for taking the HBV test for the first time were collected using a questionnaire. A total of 424 participants were enrolled (212 “healthy” controls and 212 HBV infected cases). Male sex (odds ratio [OR] = 2.08, p = 0.010), ≤ secondary education level (OR = 4.83, p<0.001), low-income level (OR = 3.79, p<0.001), rural settlement (OR = 2.17, p = 0.031), history of sexually transmitted infections (STI) (OR = 4.24, p<0.001) and ignorance of sexual partners HBsAg status (OR = 2.70, p = 0.003) all had an independent and significant association with HBV infection. Top 3 reasons for doing HBsAg test were free screening (40.3%), blood donation (15.0%) and administrative requirements (14.9%). HBV testing uptake and early detection can be improved if more sensitization and free/opportunistic screenings are implemented. A significant drop in the cost of HBV test could encourage more people to get tested.

## Introduction

Over the years Hepatitis B virus (HBV) infection has remained a major public health concern with about 296 million people living with the virus [1–3]. Although HBV shares similar transmission routes with HIV and HCV, its transmission is seemingly more feasible [4, 5]. Susceptible persons get infected during close contact with HBV infected blood or body fluids. The virus can remain stable on environmental surfaces even after decontamination with simple detergents and alcohol, further facilitating its transmission [6].

Many different transmission routes of HBV have been identified. Perinatal transmission is more common in high endemic areas while sexual transmission is more common in low endemic areas [7, 8]. The predominant HBV transmission routes in a particular community may be dependent on the economic status as well as some cultural and behavioral practices [7] of the people which predispose them to the infection. This study was aimed at identifying some demographic and behavioral factors associated with HBV infection in the study population. Effective measures to curb HBV transmission can be easily formulated or strengthened if the prevailing associated risk factors in a particular community are known. In addition to the identification and implementation of measures to reduce HBV transmission, scaling up HBV testing/screening could play a key role in eliminating hepatitis B by 2030 as envisaged by the World Health Organization (WHO) [9, 10].

The importance of HBV testing/screening cannot be overemphasized as this can help to promptly identify people at risk of contracting HBV infection and those who need counselling, monitoring/treatment which can help to prevent future burden of severe disease (liver failure, cirrhosis and hepatocellular carcinoma) [11]. A systematic review and meta-analysis conducted in Cameroon revealed an HBV prevalence of 11.2% [12]. The cumulative sample size (105,603) of this meta-analysis was relatively small compared to the actual population of the country (25 million) [13] and this could imply an overall low uptake of HBV testing in the country. Moreover, the asymptomatic nature of the disease in most cases [14] reduces the chances of suspecting it and as such, many infected individuals ignorantly live with it and may never see the need to get tested. Understanding the factors that can easily persuade someone to go in for the test in a community could help in implementing novel strategies or revising existing strategies aimed at convincing people to take the test.

## Materials and methods

### Study location, design and population

This study was conducted in the South West Region of Cameroon at the Buea Regional Hospital from March 2013 to May 2018. HBV infected individuals and “healthy” controls (≥18 years old) were enrolled as cases and controls, respectively. A “healthy” control was considered to be any person who tested negative for HBsAg, anti-HBs and anti-HBc. HBV infected individuals were considered to be anybody positive for HBsAg and anti-HBc.

### Participant enrolment

All the controls and a few HBV infected cases were enrolled during a free screening exercise (for HIV, HBV and HCV) we conducted at the Buea Regional Hospital which lasted for about 4 years. HBV infected participants were also enrolled from people who visited the hospital for various reasons (blood donations, antenatal care, medical care, consultation, etc.) and tested positive for HBsAg. The snowball sampling technique [15] was also used where already enrolled participants proposed and linked us to other eligible participants from among their acquaintances, friends and family members.

### Ethical considerations

Ethical clearance for this study was obtained from the National Ethics Committee of Research for Human Health (NECRHH) in Cameroon. Administrative authorizations were obtained from the South West Regional delegation for public health and the administrative head of the Buea Regional Hospital. Each participant signed an informed consent form at enrolment as an approval. After obtaining an assent from the individuals concerned, a consent was as well gotten from the parents/guardians of minors who took part in this study. Participants were counselled prior to testing.

### Questionnaire administration and data collection

Demographic data, behavioral risk factors and reasons for doing an HBV test for the first time were collected using an interviewer-based pre-tested questionnaire previously published elsewhere [16]. Responses on socio-economic status and behavioral patterns were assessed and grouped as reported in other studies [17] with some modifications. Monthly income level was classified into the following categories:

(i) >100,000 XAF (> 200 US dollars) per month (White-collar workers consisting of salaried service personnel, professionals and self-employed business persons) (ii) <100,000XAF (< 200 US dollars) per month (Blue-collar workers, which included agricultural, non-agricultural, skilled labourers and self-employed/salaried service personnel) and (iii) no income. Level of education was grouped into ≤ secondary and > secondary education. Age at first sexual intercourse was grouped into ≤18 years and >18years of age. The use of condom was classified into 3 different groups: never/rarely, sometimes and always. Information on the history/presence of STI was also obtained from the questionnaire.

### Sample collection, processing and laboratory analysis

Approximately 4 ml of venous blood was collected from each participant in a dry tube (no anticoagulant). The samples were allowed to clot and then centrifuged at 3000 rpm for 5 minutes to obtain serum. HBV serologic profile (panel) test was done using an immunochromatographic panel kit (Blue Cross Bio-Medical Co. Ltd, Beijing) following the manufacturer’s instructions. HBsAg and anti-HBc combined positivity was used to diagnose HBV infection.

### Statistical analysis

Data analysis was carried out using SPSS (Statistical Package for the Social Sciences, Chicago, Illinois) version 21.0. Data were presented using frequency distribution tables and summary statistics. Categorical comparisons were performed using the Pearson’s Chi-square test or the Fisher’s exact test (for 2 by 2 cells with values < 5). A logistic regression model was fitted for variables that recorded p-value ≤ 0.2 in the crude odds ratio (OR). Statistically significant differences were considered for p-values < 0.05.

## Results

A total of 424 participants were enrolled. This population comprised of 212 “healthy controls” and 212 HBV infected cases. The overall population was made up of 232 (54.7%) females and 192 (45.3%) males. The healthy control group recorded a mean age of 32.28±8.4 with ages ranging from 18 to 65 years old. The positive cases recorded a mean age of 31.21±7.5 with ages ranging from 18 to 63 years old (Table 1).

**Table 1:** Age and gender distribution of participants.

Groups (n)	Mean age ± SD (years)	Age range (years)	Gender	n (%)
HBV infected cases (212)	31.21 ± 7.5	18–63	Female	94 (44.3)
			Male	118 (55.7)
“Healthy” controls (212)	32.28 ± 8.4	18–65	Female	138 (65.1)
			Male	74 (34.9)

Males (OR = 2.08), people with ≤ secondary level of education (OR = 4.22) were significantly more associated with the infection as compared to females and people with >secondary level of education respectively. People with income level ≥100,000 XAF and people living in urban areas proved to be significantly less associated with the infection (Table 2).

**Table 2:** Demographic factors and their possible association with HBV infection.

DEMOGRAPHIC FACTORS		n	STATUS		RISK ESTIMATE					
					Crude odds ratio			Adjusted odds ratio		
			Controls	Cases	Odds ratio	95% CI*	P-value	Odds ratio	95% CI	P-value
Gender, n (%)	Male	192	74 (38.5)	118 (61.5)	2.34	1.58–3.46	**< 0.001**	2.08	1.19–3.70	**0.010**
	Female	232	138 (59.5)	94 (40.5)	1			1		
Age group (years), n (%)	<30	193	85 (44.0)	108 (56.0)	1.55	1.06–2.28	**0.025**	1.38	0.74–2.57	0.310
	≥30	231	127 (55.0)	104 (45.0)	1			1		
Monthly Income level (XAF), n (%)	No income	128	49 (38.3)	79 (61.7)	3.28	2.05–5.23	**< 0.001**	3.17	1.79–8.05	**< 0.001**
	<100,000	108	37 (34.3)	71 (65.7)	3.90	2.36–6.43	**< 0.001**	3.79	2.28–6.30	**< 0.001**
	≥100,000	188	126 (67.0)	62(33.0)	1			1		
Level of education, n (%)	≤Secondary	91	20 (22.0)	71 (78.0)	4.83	2.81–8.31	**< 0.001**	4.22	1.87–9.50	**< 0.001**
	>Secondary	333	192 (57.7)	141 (42.3)	1			1		
Marital status, n (%)	Single	219	102 (46.6)	117 (53.4)	1.41	0.96–2.07	0.079	1.59	0.99–2.18	0.094
	Married	205	113 (55.1)	92 (44.9)	1			1		
Type of school attended, n (%)	Boarding school	102	60 (58.8)	42 (41.2)	0.67	0.42–1.05	0.077	0.58	0.29–1.16	0.122
	Day school	322	157 (48.8)	165 (51.2)	1			1		
Area of residence, n (%)	Rural	133	51 (38.3)	82 (61.7)	2.32	1.53–3.54	**< 0.001**	2.17	1.32–3.40	**0.031**
	Urban	291	172 (59.1)	119 (40.9)	1			1		

*Controls*: *Healthy controls Cases*: *HBV infected individuals*.

Individuals with a history/presence of other sexually transmitted infections (gonorrhoea, syphilis, chlamydia, HIV etc) were more associated with HBV infection recording a statistically significant difference (p < 0.001) in both the crude and adjusted odds ratio (Table 3).

**Table 3:** Some behavioural factors and their possible association with HBV infection.

BEHAVIOURAL FACTORS		n	STATUS		RISK ESTIMATE					
					Crude odds ratio (OR)			Adjusted odds ratio (OR)		
			Controls	Cases	OR	95%CI	P-value	OR	(95%CI)	P-value
Age at first sexual intercourse*n (%)	≤18 years	150	51 (34.0)	99 (66.0)	2.58	1.70–3.92	**< 0.001**	1.78	1.00–3.14	0.051
	>18 years	254	145 (57.1)	109 (42.9)	1			1		
History/presence of other STIs * n (%)	Yes	56	11 (19.6)	45 (80.4)	4.64	2.32–9.28	**< 0.001**	4.24	1.75–10.29	**< 0.001**
	No	348	185 (53.2)	163 (46.8)	1			1		
Use of condom* n (%)	Rarely/never	197	93 (47.2)	104 (52.8)	0.72	0.42–1.23	0.230	0.47	0.26–0.85	**0.010**
	Sometimes	128	73 (57.0)	55 (43.0)	0.49	0.27–0.86	**0.014**			
	Always	79	31 (39.2)	48 (60.8)	1					
Ritual scarification? n (%)	Yes	229	119 (52.0)	110 (48.0)	0.84	0.58–1.24	0.381			
	No	195	93 (47.7)	102 (52.3)	1					
Roadside manicure and pedicure, n (%)	Yes	55	34 (61.8)	21 (38.2)	0.58	0.32–1.03	0.062	0.69	0.32–1.45	0.225
	No	369	178 (48.2)	191 (51.8)	1			1		
Ever had a blood transfusion? n (%)	Yes	19	7 (36.8)	12 (63.2)	1.76	0.68–4.55	0.246			
	No	405	205 (50.6)	200(49.4)	1					
Tattoo, n (%)	Yes	8	3 (37.5)	5 (62.5)	1.68	0.40–7.13	0.480			
	No	416	209 (50.2)	207 (49.8)	1					
Knowledge of sexual partner’s HBV status* n (%)	Knows partner is Positive	80	58 (72.5)	22 (27.5)	0.28	0.17–0.49	**< 0.001**	0.37	0.20–0.71	**0.003**
	Don’t know/knows partner is Negative	324	139 (42.9)	1 185 (57.1)	1			1		
Taken HBV vaccine? n (%)	No	393	t 191 (48.6)	20 202 (51.4)	2.22	1.02–4.84	0.045	1.19	0.24–2.11	0.153
	Yes	31	21 21 (67.7)	10 (32.3)	1			1		

*People who have never had sex are excluded Controls: *healthy controls Cases*: *HBV infected individuals*.

People who knew that their sexual partners were HBsAg positive were significantly less associated with the infection as compared to those who did not know their sexual partner’s status and those who knew their partners were HBsAg negative. This observation was statistically significant in both the crude and adjusted odds ratio (Table 3).

**Fig 1:**
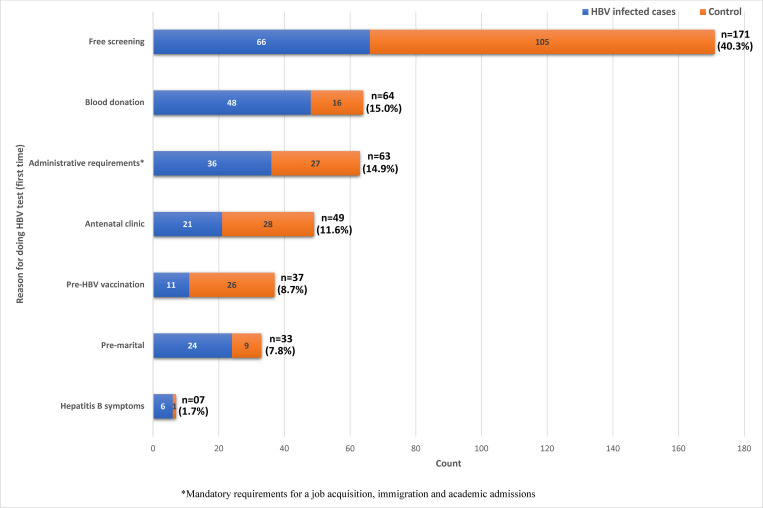
Reasons for first ever HBV testing for all participants (n = 424).

The 3 main reasons for getting tested for the first time were free screening (40.3%), Blood donation (15.0%) and administrative requirements (14.9%) as seen in Fig 1. Overall, only 7 (1.7%) participants admitted doing the test because they developed some symptoms common in HBV infection and the doctor requested for the test. One hundred and seventy-one participants admitted doing HBV test for the first time during free screening exercises. Of these 171 participants, 93 (54.4%) admitted being screened for the first time by us.

Average age at first HBV testing was highest for those who tested following pre-marital recommendations (32.1±6.4 years). Age range at first HBV testing was widest (16–65 years) for those who tested for the first time during free screenings (Table 4).

**Table 4:** Distribution of gender and age at first HBV testing across various testing reasons.

Reason for first HBV testing (n)	Gender	n(%)	Age at first HBV testing (years)			
			Mean ± SD	Range	Median	IQR*
Free screening (171)	Female	114(66.7)	29.8 ± 7.8	16–65	29.0	11
	Male	57 (33.3)				
Blood donation (64)	Female	14 (21.9)	26.8 ± 7.7	20–41	25.0	10
	Male	50 (78.1)				
Sickness (7)	Female	3 (42.9)	26.3 ± 8.0	15–39	26.0	19
	Male	4 (57.1)				
Pre-vaccination (37)	Female	20 (54.1)	30.5 ± 9.40	18–54	30.0	12
	Male	17 (45.9)				
Pre-marital (33)	Female	13 (39.4)	32.1 ± 6.4	22–43	32.5	7
	Male	20 (60.6)				
Administrative (63)	Female	9 (14.3)	26.1 ± 5.54	20–40	23.0	8
	Male	54 (85.7)				
Antenatal (49)	Female	49 (100)	26.1 ± 5.04	18–37	25.0	7
	Male	-				

*Interquartile range.

Only 6 (2.8%) of the HBV infected individuals got to know their status because of ill health (probable presentation of some signs and symptoms which made the doctor to demand HBsAg test). Forty-eight (22.6%) of them got to know their status because they wanted to donate blood (Fig 1). Free screening accounted for 31.4% of all the identified HBV infected cases in our study.

## Discussion

Our study showed that males were more associated with HBV infection as compared to females. Previous studies have established this [14, 18, 19] and could be attributed to hormonal and some protein expression differences. Oestrogen has been shown to play a protective role in females at reproductive age [20–22]. Some particular unusual apolipoprotein (A-I) found only on the hepatocytes of males has been shown to predispose them to the infection and its associated complications [23].

People with no income or a monthly income of <100,000XAF were more associated with HBV infection as compared to people with ≥ 100,000XAF monthly income as seen in another study [24]. It is an established fact that high income earners are less associated with infectious diseases as they can have access to a more convenient life style [25] and afford preventive measures like vaccines [26, 27]. The cost of the vaccine still stands as a major challenge in controlling the spread of the infection in poor countries [28]. Low income is a major characteristic of people living in rural settings both in Cameroon [29] and other parts of the world [30–33]. Our study also showed that people who live in rural areas were significantly more associated with HBV infection compared to those in urban areas as seen in other studies [12, 34–36]. Poverty, high level of illiteracy, limited knowledge on preventive care, poor access to health care and dearth of sensitization may all account for the poor health outcomes in rural areas [33, 37].

Educational status below secondary level was an independent and significant predictor of HBV infection in our study. Some other studies [38–40] have recorded similar findings. People with higher educational attainment tend to live a healthier live as compared to those with poor educational background [41]. Integrating routine comprehensive health talks on HBV infection in primary and secondary schools could help to curb the transmission of HBV in our community as this would guarantee youth awareness of the infection and its preventive measures during the early stages of their academic lives.

As reported in other studies [42, 43], individuals with a history of or presence of other sexually transmitted infections (STI) were significantly associated with HBV infection. This was also noticed in other studies. Despite all the identified sexual risk behaviours, our study instead showed that people who always use condoms were more associated with HBV infection. Some of the participants who acknowledged consistent use of condom also admitted having contracted at least one STI (chlamydia infection, gonorrhoea, syphilis etc) before in their lives. This gave us every reason to doubt their responses and knowledge on proper use of condoms. Unfortunately, our study did not assess knowledge and practise of proper use of condoms. It is worth noting that assessment of sexual risk behaviours is usually very challenging because some people tend to be very reserved and shy when it comes to presenting the truth about their sexual life even for research purposes where there is a guarantee of confidentiality [44, 45]. Interestingly, we realised that participants who knew their sexual partners were positive for HBV infection were independently and significantly not associated with the infection as compared to those who knew their partners were negative and those who did not know the status of their sexual partners. This highlights the relevance of status disclosure in preventing diseases transmitted through close or sexual contact. It is possible that people who knew their partners were HBsAg positive probably took adequate precautions like getting vaccinated, practising safe sex etc to ensure that they do not contract the infection themselves over time. Perhaps individuals who did not know their partners’ HBV status probably paid less attention to relevant preventive measures.

History of blood transfusion was not associated with HBV infection in this study. This was observed in other studies as well [18, 46–48] but a study conducted in the North West Region of Cameroon [39] found an association. As a result of better diagnostic and screening methods of Transfusion Transmitted Infections (TTI) prior to blood transfusion, HBV transmission via this route seems to be decreasing over time [49]. The careful and meticulous screening of donor’s blood usually disqualifies many donors due to HBsAg positivity. Our study further throws more light on this as blood donation was recorded as the second most common reason why our participants got tested for the first time and this exercise alone detected HBV infection in 48 (22.6%) of the 212 HBV infected participants.

The third most common reason for HBV screening in this study was subjection to some obligatory administrative requirements (14.9%). This includes requirements for a job acquisition, immigration, academic admissions etc. If these obligatory testing requirements where not applicable, some of the people in this particular group could have still been ignorant of their status. Therefore, obligatory testing as an administrative requirement can improve uptake of HBV testing. However, because most of such institutions are not healthcare institutions, we cannot guarantee if they consider linking people to care (for those who test HBsAg positive) or vaccine acquisition (for those who test HBsAg negative) after testing as per the recommendations of WHO [50].

Less than ten percent (8.7%, n = 37) of our study participants got tested for the first time as prerequisite for HBV vaccination. HBV pre-vaccination testing (HBsAg or anti-HBc) is usually recommended for people in high risk groups (healthcare workers, sexual and household contacts of HBV infected persons etc) and for foreigners born in HBV endemic regions as per CDC guidelines [51]. It could be quite important for this recommendation to as well include natives of HBV endemic regions like Cameroon as this would help improve uptake of HBV testing as well as identify those who do not need the vaccine because of a current infection or presence of an already developed natural immunity to the infection. Identifying such people prior to vaccination can be cost effective on their part as they would not have to pay for a vaccine they don’t need. The cost of taking the complete vaccine is way more expensive than the cost of doing the test in Cameroon.

Only seven (1.7%) participants got tested for the first time due to clinical suspicion of HBV infection. The long-term asymptomatic nature of the disease greatly influences the number of people who end up getting tested as medical doctors seldom request for the test. As such, many infected cases go undetected and their ignorance could promote disease progression and further transmission to other uninfected susceptible people. To improve the uptake of HBV testing in healthcare settings, the measures from provider-initiated HIV testing strategy could be replicated as a form of opportunistic screening [52] at various entry points, even in the absence of corroborative signs and symptoms. Considering the fact that HBV is like 3 times more prevalent (11.2%) [12] than HIV (3.7%) [53] in our population and also the fact that HBV and HIV infection are both major public health problems, we propose and encourage opportunistic screening for HBV infection in healthcare settings in Cameroon.

In this study, voluntary participation in a free screening exercise was the most common reason for first-time tester with over fifty percent (54.4%) across the widest age range (16–65 years). The significant proportion from our free screening was quite evident because the exercise lasted for long (about 4 years) and as such, could cover more people over time. However, the “free screening exercise” pretext as a reason for HBV testing may have been overrated in this study because our screening period just might have presented itself at a time when some participants realised the importance of doing the test for various reasons (pre-marital, pre-vaccine etc) but chose not to disclose these reasons to us. Another issue worth addressing here is the cost of doing HBsAg test. Although HBV infection happens to be more prevalent as compared to HIV in our community, HBsAg test is more expensive than HIV test. “Free screening exercises” are usually free of charge and this aspect alone is a huge motivation for people to voluntarily subject themselves to it. Considering the fact that Cameroon is classified as a lower middle-income country according to world bank and also the fact that our study revealed an association between low income and HBV infection, a practical approach to detect more HBV infected people in our community could be to lower the cost of the test significantly or to offer the test free of charge.

This study had some limitations. The findings could have been more representative of the reality in our study area if the sample size was larger. This study was strictly a hospital-based setting. A hospital/community-based setting could have been ideal for this research. The use of a confirmatory test would have added more value to our diagnosis.

In conclusion, male sex, low-income level, rural settlement, ≤ secondary level of education, history of STI and ignorance of sex partners’ HBV status were all significantly associated with HBV infection. Free screening, blood donation and administrative requirements were the most common reasons for HBV testing in our study population. Sensitization even at the level of primary and secondary schools could help educate the population early enough to prevent transmission. Uptake of HBV testing and early detection can be improved if more (prolonged) free/opportunistic screenings are carried out and if the health sector subsidizes the cost of the test significantly as a way to encourage people to get tested.

## Supporting information

S1 Data Raw data files. (XLSX)
